# Aneurysmal systemic-to-pulmonary arterial connection mimicking MAPCA in a structurally normal adult: computed tomography angiography diagnosis and endovascular exclusion

**DOI:** 10.1186/s43044-026-00749-0

**Published:** 2026-05-21

**Authors:** Saubhagya Dhakal, Nikahat Yasmine, Nevzat Karabulut

**Affiliations:** Department of Radiology, University Of Iowa hospital and clinics, Iowa City, USA

**Keywords:** Systemic-to-pulmonary arterial connection, Major aortopulmonary collateral artery, MAPCA, Bronchial artery aneurysm, Isolated systemic arterial supply to normal lung, Computed tomography angiography, Thoracic endovascular aortic repair, Coil embolization

## Abstract

**Background:**

Major aortopulmonary collateral arteries (MAPCAs) are anomalous systemic-to-pulmonary vessels classically associated with complex congenital heart disease. In adults with structurally normal hearts, however, anomalous systemic-to-pulmonary arterial connections represent a broader diagnostic spectrum that includes MAPCA-like vessels, bronchial or non-bronchial systemic arterial collaterals, and isolated systemic arterial supply to normal lung (ISSNL). Aneurysmal degeneration in this setting is uncommon and clinically important because of the risk of rupture or hemorrhage.

**Case presentation:**

We report a 48-year-old man presenting with acute non-exertional chest pain. Computed tomography angiography (CTA) demonstrated a 2.6-cm saccular aneurysm arising from a tortuous systemic artery originating from the proximal descending thoracic aorta and extending toward the left lower lobe pulmonary arterial circulation. CTA delineated the vascular origin, course, aneurysmal morphology, and absence of associated congenital cardiac abnormalities. Digital subtraction angiography confirmed a single systemic-to-pulmonary arterial communication. The patient underwent thoracic endovascular aortic repair with adjunctive coil embolization, resulting in complete aneurysm exclusion and symptom resolution.

**Conclusions:**

This case highlights an aneurysmal systemic-to-pulmonary arterial connection with MAPCA-like features in a structurally normal adult. The case emphasizes the importance of CTA in differentiating MAPCA-like vessels from bronchial artery aneurysm, non-bronchial systemic collaterals, and ISSNL, and demonstrates the effectiveness of endovascular therapy in excluding a potentially high-risk aneurysmal vascular lesion.

## Introduction

Major aortopulmonary collateral arteries (MAPCAs) are abnormal systemic arteries that provide pulmonary blood flow in patients with congenital heart disease, particularly pulmonary atresia and tetralogy of Fallot [1–3]. These vessels arise from persistent embryologic systemic-to-pulmonary arterial connections that normally regress during development of the sixth aortic arch and native pulmonary arteries [4]. MAPCAs are well recognized in neonates and infants with complex congenital heart disease; however, systemic-to-pulmonary arterial connections identified in adults without structural cardiac abnormalities represent a broader and more heterogeneous group of vascular entities.

In adults, anomalous systemic arterial supply to the lung may include MAPCA-like vessels, bronchial artery hypertrophy or aneurysm, non-bronchial systemic collaterals, and isolated systemic arterial supply to normal lung (ISSNL) [5–8]. These entities can overlap in imaging appearance but differ in embryologic origin, pathophysiology, and clinical implications. Therefore, precise anatomic characterization and cautious terminology are essential, particularly when native pulmonary arterial anatomy is preserved and no congenital cardiac lesion is present.

Aneurysmal transformation of a systemic-to-pulmonary arterial connection is uncommon but clinically important because of the potential risk of rupture, hemoptysis, mediastinal hemorrhage, or other acute complications [7, 9–12]. We report a case of an aneurysmal systemic-to-pulmonary arterial connection with MAPCA-like features in a structurally normal adult presenting with acute chest pain, diagnosed by computed tomography angiography (CTA), and successfully treated with thoracic endovascular aortic repair (TEVAR) and adjunctive coil embolization.

## Case presentation

A 48-year-old man with no prior cardiovascular or pulmonary history presented with acute non-exertional chest pain. He denied dyspnea, hemoptysis, syncope, palpitations, or fever. There was no known history of congenital heart disease. Electrocardiography demonstrated normal sinus rhythm without ischemic changes. Cardiac biomarkers and D-dimer levels were within normal limits. Transthoracic echocardiography revealed a large aneurysmal vascular structure adjacent to the proximal descending thoracic aorta. Contrast-enhanced CTA of the chest was subsequently performed to exclude pulmonary embolism or acute aortic pathology.

**Fig. 1:**
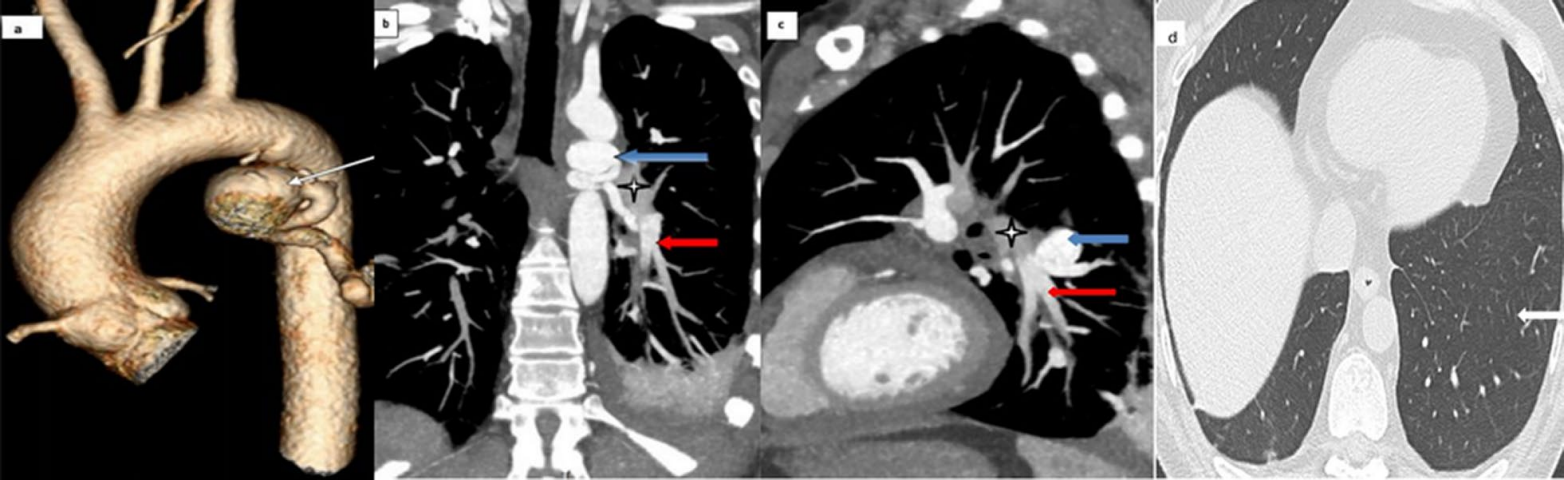
CTA demonstrating an aneurysmal systemic-to-pulmonary arterial connection with MAPCA-like features. **a** Coronal volume-rendered reconstruction shows a tortuous systemic vessel arising from the descending thoracic aorta with focal saccular aneurysmal dilatation (white arrow). **b** Coronal maximum-intensity projection image acquired in the aortic phase demonstrates the aneurysmal systemic-to-pulmonary vessel (blue arrow) extending toward the left lower lobe pulmonary arterial circulation (red arrow). Relative lack of opacification in the proximal left pulmonary artery (asterisk) is favored to reflect flow-related preferential systemic opacification rather than definitive absence. **c** Sagittal maximum-intensity projection image depicts the aneurysmal systemic-to-pulmonary vessel (blue arrow) and its communication with the pulmonary arterial circulation (red arrow). Relative lack of opacification in the proximal left pulmonary artery is again noted (asterisk). **d** Axial lung-window image demonstrates normal pulmonary parenchyma in the left lower lobe without consolidation or bronchiectasis (white arrow)

**Fig. 2:**
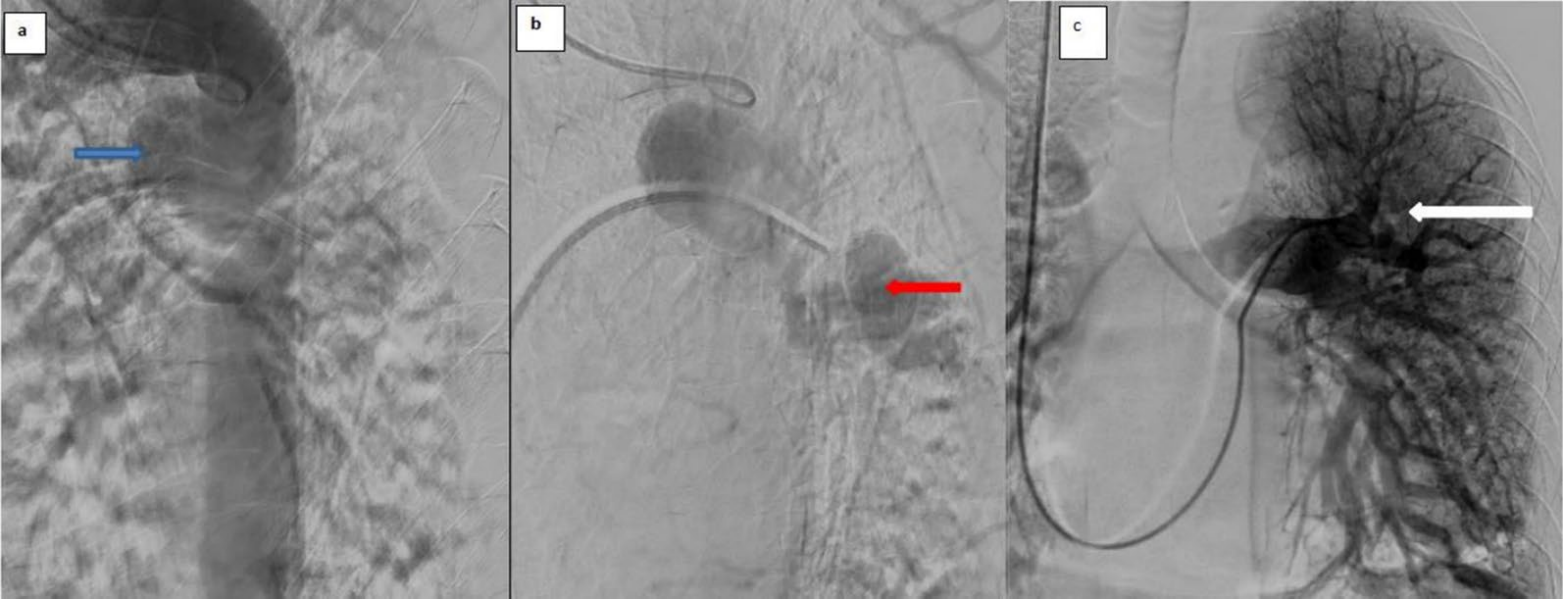
Digital subtraction angiography confirming systemic-to-pulmonary arterial communication. **a** Angiographic image demonstrates the aneurysmal systemic-to-pulmonary arterial connection. **b** Angiographic image shows extension of the anomalous systemic vessel toward the left lower lobe pulmonary arterial circulation. **c** Pulmonary angiogram shows opacification of the pulmonary arterial vasculature without direct visualization of the anomalous systemic vessel, supporting the need for multimodality correlation

CTA demonstrated a markedly dilated and tortuous systemic vessel arising from the proximal descending thoracic aorta, with a focal proximal saccular aneurysm measuring 2.6 cm (Figs. 1 and 2). The vessel coursed inferiorly toward the left lower lobe pulmonary arterial circulation, establishing a systemic-to-pulmonary arterial communication. No pulmonary atresia, ventricular septal defect, patent ductus arteriosus, or other structural cardiac abnormality was identified. The native pulmonary arteries were otherwise preserved. Relative lack of opacification in the proximal left pulmonary artery on the aortic-phase images was interpreted as likely flow-related preferential systemic opacification rather than definitive absence of the pulmonary artery; this finding was considered important in the differential diagnosis.

**Fig. 3:**
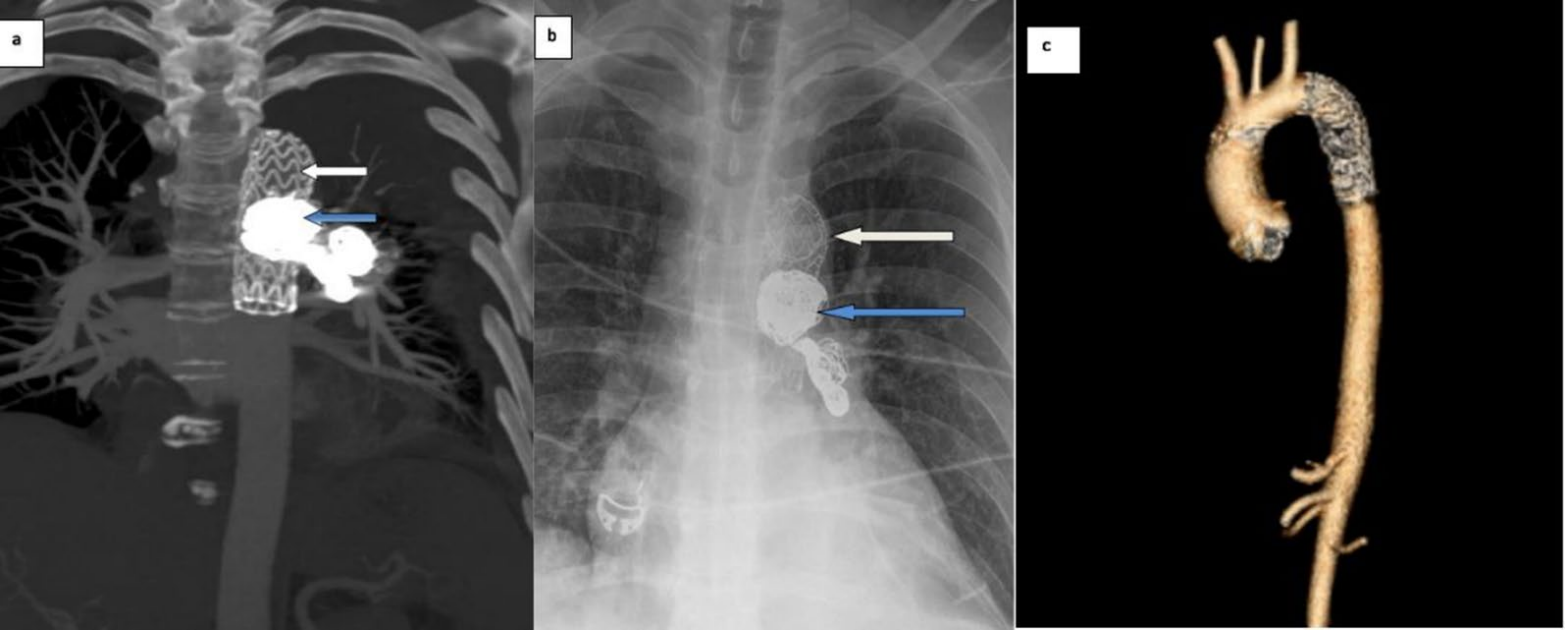
Post-intervention imaging demonstrating successful aneurysm exclusion following thoracic endovascular aortic repair (TEVAR) with adjunctive coil embolization. **a** Coronal multiplanar reconstruction CT image shows the endovascular stent graft within the thoracic aorta (white arrow) and embolization coils within the anomalous systemic-to-pulmonary vessel (blue arrow). **b** Chest radiograph demonstrates the thoracic endovascular stent graft and embolization coils. **c** Follow-up angiographic volume-rendered image shows absence of contrast opacification of the previously aneurysmal systemic-to-pulmonary vessel, consistent with successful treatment

Digital subtraction angiography confirmed a single systemic-to-pulmonary arterial communication arising from the descending thoracic aorta (Fig. 2). Given the aneurysmal morphology and potential risk of rupture, a multidisciplinary decision was made to proceed with endovascular exclusion. The patient underwent TEVAR with adjunctive coil embolization of the anomalous systemic-to-pulmonary vessel. Coil embolization was used to occlude the collateral vessel, and thoracic endovascular stent-graft placement was performed to exclude the aortic origin and eliminate persistent aneurysm pressurization. Post-procedural imaging demonstrated complete exclusion of the aneurysm without residual shunt (Fig. 3). The patient experienced complete symptom resolution after the procedure and remained asymptomatic at 3-month follow-up.

## Discussion

MAPCAs are most frequently encountered in pediatric patients with congenital heart disease and diminished pulmonary blood flow [1–3]. Their embryologic basis involves persistence of primitive systemic-to-pulmonary arterial connections that fail to regress during normal development [4]. In this classic setting, MAPCAs serve as essential sources of pulmonary perfusion, particularly in patients with pulmonary atresia or major pulmonary arterial abnormalities.

In contrast, systemic-to-pulmonary arterial connections identified in adults with structurally normal hearts represent a broader and more heterogeneous group of vascular entities. These include bronchial artery enlargement or aneurysm, non-bronchial systemic collaterals, and variants such as ISSNL [5–8]. Distinguishing among these entities is clinically relevant because they differ in embryologic origin, pathophysiology, expected pulmonary arterial anatomy, and implications for management.

The present case demonstrates an aneurysmal systemic artery arising from the descending thoracic aorta and extending toward the left lower lobe pulmonary arterial circulation, with no associated congenital cardiac abnormality. While the morphology and direct systemic-to-pulmonary communication resemble descriptions of MAPCAs, several features—including the absence of congenital heart disease and the presence of otherwise preserved pulmonary arterial anatomy—raise the possibility that this lesion represents a non-bronchial systemic arterial supply or variant collateral pathway rather than a classic embryologic MAPCA.

Accordingly, this case is best interpreted as an aneurysmal systemic-to-pulmonary arterial connection with MAPCA-like features, highlighting the spectrum and diagnostic ambiguity of these vascular anomalies in adults. This distinction is not merely semantic, as it reflects differences in developmental origin and clinical context. Importantly, regardless of classification, aneurysmal transformation carries significant clinical implications because of the risk of rupture, hemoptysis, or mediastinal hemorrhage [7, 9–12].

A structured differential diagnosis is essential in this setting. ISSNL represents a rare entity within the sequestration spectrum, characterized by an anomalous systemic artery supplying otherwise normal lung parenchyma without associated bronchial or parenchymal developmental abnormality [6]. It classically involves an artery arising from the descending thoracic aorta and supplying the basal segments of the left lower lobe, and it may mimic other systemic-to-pulmonary vascular connections [5, 6]. Bronchial artery aneurysm is another important consideration, particularly when systemic arterial aneurysmal dilatation is associated with bronchopulmonary shunting [7]. Bronchial artery aneurysms are rare but clinically important because rupture risk may be present irrespective of aneurysm size, and prompt recognition and treatment are generally recommended [7].

CTA played a central role in narrowing this differential diagnosis. Multidetector CTA can define vessel origin, course, aneurysmal morphology, parenchymal distribution, bronchial anatomy, and native pulmonary arterial supply, all of which are necessary for distinguishing MAPCA-like vessels from bronchial or non-bronchial systemic collaterals and ISSNL [5, 6, 8]. Multiplanar and three-dimensional reconstructions are particularly valuable in defining the spatial relationship of these vessels to the thoracic aorta and pulmonary arteries. Digital subtraction angiography remains important for confirmatory evaluation and for endovascular treatment planning.

The mechanism of aneurysm formation in this case is likely related to chronic exposure of an anomalous systemic artery to systemic arterial pressure. Persistent high-flow conditions, particularly in tortuous vascular segments or near the aortic origin, may result in focal wall stress, progressive weakening, and saccular dilatation. Unlike cases associated with pulmonary hypertension or increased pulmonary vascular resistance, our patient demonstrated no imaging evidence of pulmonary arterial enlargement or elevated pulmonary pressures, suggesting that localized hemodynamic factors were likely contributors to aneurysm formation.

Clinically, the patient's presentation with acute chest pain is plausibly explained by aneurysmal wall stress or stretching of adjacent mediastinal structures. Although such vascular anomalies may remain asymptomatic for extended periods, aneurysmal degeneration substantially increases the risk of acute complications. The delayed presentation into adulthood may reflect gradual hemodynamic adaptation and progressive enlargement over time before reaching a symptomatic threshold.

An additional observation in this and previously reported cases is involvement of the left lower lobe. Although the reason for this predilection is not definitively established, it may relate to embryologic persistence of segmental systemic arterial supply in the lower lobes or regional hemodynamic factors favoring collateral development in dependent lung regions [5, 6].

From a management perspective, endovascular therapy represents an effective and minimally invasive approach for excluding aneurysmal systemic-to-pulmonary arterial connections. Techniques including coil embolization, liquid embolic agents, vascular plug placement, and thoracic endovascular stent-graft exclusion have been described in selected systemic-to-pulmonary vascular lesions [6, 7, 10, 11]. In the present case, combined TEVAR and adjunctive coil embolization achieved complete aneurysm exclusion by addressing both the aortic origin and collateral vessel flow. Compared with open surgical treatment, endovascular management may reduce procedural morbidity while providing definitive therapy in appropriately selected patients.

This case has limitations. First, classification of the lesion as a true embryologic MAPCA cannot be established with absolute certainty because of overlap with ISSNL, bronchial artery aneurysm, and non-bronchial systemic collateral pathways. Second, the interpretation of proximal left pulmonary arterial opacification is limited by single-phase CTA timing and flow dynamics. Third, follow-up remains short, and longer-term imaging would be useful to confirm durable exclusion and absence of recanalization.

Overall, this case underscores the importance of recognizing aneurysmal systemic-to-pulmonary arterial connections in adults, particularly when presenting as atypical thoracic vascular lesions. It also highlights the diagnostic challenge of distinguishing MAPCA-like vessels from other systemic arterial variants in the absence of congenital heart disease and emphasizes the pivotal role of CTA in diagnosis, differential consideration, and procedural planning.

## Conclusion

An aneurysmal systemic-to-pulmonary arterial connection with MAPCA-like features in a structurally normal adult is an uncommon but clinically important vascular lesion. CTA is crucial for defining vascular anatomy, assessing native pulmonary arterial supply, excluding associated congenital cardiac abnormalities, and planning endovascular treatment. Careful differentiation from ISSNL, bronchial artery aneurysm, and non-bronchial systemic collaterals is essential. Timely endovascular intervention with TEVAR and coil embolization can provide safe and effective aneurysm exclusion and may prevent potentially catastrophic complications.

## Data Availability

No datasets were generated or analysed during the current study.

## Abbreviations

MAPCA: Major aortopulmonary collateral artery
CTA: Computed tomography angiography
TEVAR: Thoracic endovascular aortic repair
CT: Computed tomography
ISSNL: Isolated systemic arterial supply to normal lung
BAA: Bronchial artery aneurysm
